# The Book World of Medicine and Science

**Published:** 1896-08-15

**Authors:** 


### The Book World of Medicine and Science.

Strathpeffer Spa: Its Waters and Climate. By R. Fortescue Fox, M.D.Lond. (Adam and Charles Black. 1896.)

This useful treatise gives a careful and elaborate account of the valuable mineral waters at Strathpeffer, Ross-shire, N.B., with a copious analysis of the waters and an account of the climatic conditions of the place and neighbourhood. As a guide to the medical profession it will prove of great value, and bears convincing testimony to the health-giving properties of the waters, especially in cases of gout and rheumatism and other kindred diseases. We strongly recommend any who may be seeking, under medical advice, restoration to health from nature's healing springs to read the book for themselves. The country is beautiful and mountainous, and the place easy of access. A chapter is devoted to the geological and botanical features of the district, while the appendix, to which is annexed a useful map, furnishes valuable information as to the various places of interest in the neighbourhood. We would suggest that in the next edition some details might be usefully given as to the accommodation afforded by hotels, houses, and lodgings, with the tariff, &c.

Proceedings of the American Medico-Psychological Association at the Fifty-First Annual Meeting, Denver, 1895. Pp. 258.

While it could hardly be said that this volume is particularly valuable from a scientific point of view, it is evidence of activity and zeal in the cause of medical treatment of the insane which might advantageously find an echo on this side of the Atlantic. The volume is a collection of papers read at the 1895 meeting of the association, and it is further a sort of directory of American Alienist Physicians. The president's address is both interesting and instructive. Dr. Cowles shows how essential unity of control is to successful administration of large institutions, and he insists that this control should be vested in the medical superintendent. He quotes the words of the poet Whittier that "an institution is the shadow of a man." Dr. Wise's paper on medical work in the wards is an eminently practical one, and the following passage will show how like American asylums are to our own, in some respects at all events. "Allowing that insane hospital physicians, as a class, are superior men, and those who gain advancement are selected men, it must be admitted that sometimes the temptation of a hundrum daily routine is not resisted, and they become medical automatons. Occasionally a brilliant, active, and sanguine assistant bent on original research will flame across the medical firmament of a hospital, but too frequently like a comet without an orbit. In other instances, with a definite goal, and with loyalty to well-matured and precise methods, the assistant develops into the scientist, and his department becomes not only a hospital in name but in fact." Dr. Wise thinks it is a mistake to separate the pathological and clinical work, and he holds that the appointment of a special pathologist is to be deprecated, a principle which has more than once been enunciated in the columns of this journal. Dr. Pilgrim descants on the dietary of the State hospitals for the insane, and incidentally refers to the use of large dining halls. "A long experience," he says, "with associated dining rooms, where large numbers are obliged to dine together, has made me unalterably opposed to them." And when "the fastidious man must sit where he can see the glutton gorge, and the timid melancholic may be startled by the piercing cry of the epileptic, the food will go untasted and the strength will wane." We see plenty of this in England. Large associated dining halls, as they are called, have not one argument in their favour, and yet year after year our asylums are built with these adjuncts, and protest seems useless. Dr. Rohé contributes a paper on pelvic disease in women. He discusses the point whether any operation can be legally performed on a lunatic. According to the Pennsylvanian Committee in Lunacy, a surgical operation upon a lunatic is in all cases "illegal and unjustifiable." Dr. Rohé has failed to find any statutory enactment governing the authority as to surgical operations; but he takes the practical, sensible view that if the operation be for the good of the patient and performed in good faith, it would be right to undertake it. Nevertheless, we fear that in a strictly legal sense the lunatic could not himself give consent, and there does not seem any law whereby the lunatic's friend or his medical attendant can decide for him. Dr. Clarke gives details of five cases of chronic insanity treated by thyroid feeding. He points out that the treatment requires most judicious handling, being as dangerous in some cases as it is beneficial in others. "Cell nutrition is undoubtedly affected in a striking manner, increased metabolism occurs as a result of a quickened circulation, and the antotoxic process that exists in some, if not all, cases of mental disease, is interfered with in a way that may be beneficial. In other words, some patients are given a new start. On the other hand, if the vitality is low and the patient has not the ability to recover from the fever, decided harm will result, and a rapid decline in strength probably takes place." Dr. Brush follows the same subject up with careful details and charts of six cases. In one of these decided benefit seemed to be induced by the thyroid, and in two others improvement followed. Other papers are by Drs. Munson, Richardson, Clark, Meyer, Eyman, Bannister, Berkley, Mitchell, and Wanghop.

The Biological Problem of the Day. By Oscar Hertwig. Translated by P. Chalmers Mitchell, M.A. (Scientific Handbooks. W. Heinemann, 1896.)

At the present time everybody likes to hold some views and to have something to say on the biological problem which forms the subject of this essay; but whether they accept the "preformative theory" of Weismann, or, with Hertwig, criticise his conceptions or deductions, intelligent readers will be grateful to Mr. Mitchell for his translation of this important treatise and for his explanatory introduction, since, by reading them, they will be enabled to formulate more concrete ideas, and express their views with a sense of greater assurance. Closely associated with the fundamental hypothesis of development dealt with in these pages are the kindred questions of the inheritance of acquired characters, and the so-called maternal impressions; but since a clear understanding of these two latter questions involve an intelligent conception of the general theories of development, the class of readers who will read Hertwig's essay with interest will include representatives of professions as widely divergent as those of biology and nursing. Although this essay is rather a criticism of Weismann's views than an exposition, taken in conjunction with Mr. Mitchell's introduction, the reader will obtain a better insight into its general features than is obtainable from other sources available to those who can only read English. In addition, the fallacies and weaknesses of Weismann's theory are abundantly explained by Hertwig, who, though disclaiming all "love of polemics," enters with sufficient spirit into the controversy to infuse an agreeable amount of excitement into the reading. Fascinating though the two rival theories undoubtedly are, after perusal and digestion of the arguments presented in the volume, the average reader will probably conclude that Weismann's views may be visionary, and that "they cannot encounter concrete investigation," but that Hertwig's theory is little less visionary, and little more amenable to scientific demonstration.

